# Manitoba Veterinary Medical Association

**Published:** 1896-06

**Authors:** 


### MANITOBA VETERINARY MEDICAL ASSOCIATION.

(Concluded from page 158.)

### Remarks of Dr. R. Price, of St. Paul, Minn., and Dr. Rutherford, of Portage la Prairie, on the subject of "Tuberculosis," at the meeting held December 17, 1895.

Dr. Price: I came here entirely unprepared to meet you or say anything on veterinary matters, but am very glad to have met so many practitioners holding positions of honor and filling them with ability. It was a surprise to learn that your city has provision for inspection of dairy herds and testing them for tuberculosis, and that it is being so thoroughly carried out by Dr. Hinman, who has been appointed to the position of City Veterinary Inspector. When we know that the disease is a very ancient one, and was recognized in both mankind and cattle for centuries, it is a wonder, to all progressive men, that no stringent methods have been previously adopted for its suppression. In the slaughter-houses of the city of St. Paul I have watched with interest the method adopted by the Jews for slaughtering, which dates back to Moses. Examination is made, previous to the removal of the lungs, for any adhesion between costal and visceral pleuræ. Should there be even a slight band joining them, the same is carefully cut, the lungs removed, their surface examined for any pleural detachment; the lungs are next inflated by an assistant, when should any air escape from the lungs and appear under the pleura the carcass is condemned and sold to the Christians in places where there is no meat or live-stock inspection, showing that the danger to the Jewish race from consumption of such flesh has long been recognized. The Jews, however, pass "lumpy jaw" and tabes mesenterica, apparently not recognizing either disease. When we see that mortality tables acknowledge a death-rate of one in every seven from phthisis, we realize how prevalent the disease is, yet people have become so accustomed to hearing said, "died from consumption," that it produces no sense of fear, whereas were it smallpox or cholera that occasioned so many deaths the greatest excitement would prevail and every effort be put forth to suppress the disease. It is like the natives of Mecca, Arabia, where cholera exists endemically; they pay little attention to it and make no effort to suppress the malady. Yet we, who are better educated and supposed to be more intelligent, treat tuberculosis like they do cholera. We say, "It is the will of God," and so let it go. I for one do not believe it is, but that we are endowed with intelligence, and that we should use it to overcome these obstacles to life and health.

In the city of New York the percentage of cattle affected ran as high as 98, the lowest being 5. Is it a wonder that infants whose principal diet is cow's milk should suffer from bowel troubles, with a large mortality therefrom. In the State of New York, out of 20,000 cattle tested, 3½ per cent. reacted to the tuberculin injections. These tables show that where animals are crowded together in an impure atmosphere, with poor ventilation, the disease spreads most rapidly. Experiments in Germany by the most eminent pathologists have proved that the bacilli are not destroyed by the gastric juice; and the late Royal Commission appointed to investigate the disease in England reports that cooking is not always sure to kill them; that large joints are not penetrated sufficiently by the heat; and that their centres contained living germs of tuberculosis. It is now universally admitted by all prominent authorities that milk may contain the bacilli, even when the udder is not affected, and that such is dangerous to mankind. Even the knives used in butchering or cutting up raw meat will convey the germs of tubercle from a diseased to a healthy piece.

In making our autopsies insufficient time and care are taken in carrying them out fully, often resulting in failure to find lesions, except when of an extensive character, and animals are pronounced healthy. Whereas, if carefully conducted, numerous glands would be found containing tubercles, and even the lungs may have small ones after being passed over as sound. At the Minnesota State experiment farm Dr. Reynolds, who has charge of the Veterinary Department, is conducting a series of experiments on cattle, rabbits, and rats. He selected eight head of grade cattle that had reacted to tuberculin, and at regular intervals repeated the test. He found that after a time reaction ceased. As he will himself report fully the results of these experiments, I will now merely refer to them. At post-mortems held on some of the cows, a couple showed very extensive pleural and lung changes, others only slight ones. Whereas one that had given a decided reaction was apparently free from evidences of tuberculosis, yet on making a more careful search a caseous nodule was found in one lung, and a bronchial and tracheal gland contained undoubted evidences of the disease, which microscopical examination confirmed.

As to the use of such flesh for human food in this country, where meat is plentiful and cheap, there is no necessity for taking chances; and all diseased animals or carcasses should be condemned and rendered, no matter how slight the lesion is. No progressive pathologist who has any respect for his reputation will state that an animal can be affected with a contagious disease and the flesh be perfectly healthy. Who can positively state the extent to which the germs are disseminated throughout the system, and how much toxin the flesh contains? As cooking has no effect on these toxins, which are poisons, their ingestion cannot under any condition be healthful.

The sooner the disease is stamped out and all fresh arrivals carefully examined and tested, the smaller the loss will be to the Province. We all know with what fearful rapidity it spreads under favorable conditions in herds when even a single diseased beast is introduced into it, and the terrible losses it entails to the owner. I look forward with extreme interest to the results of your present effort to eradicate tuberculosis from the dairy herds, and feel certain that when this is done the mortality among our little ones will be enormously reduced, as well as the general health of the community improved.

Dr. Rutherford, of Portage la Prairie, followed. He said that it was unfortunate that so few people appeared to realize the danger to human life arising from the use of the meat and milk of animals suffering from tuberculosis. It was still more unfortunate that there were to be found in the ranks of both the medical and the veterinary professions men who, either from ignorance of the subject or from a desire to allay the fears of the public, were placing themselves on record as opposed to the thorough inspection of dairies and slaughter-houses. That a very grave danger existed in the use of the milk from tuberculous cows and the meat of animals suffering from generalized tuberculosis was surely patent to anyone who took an interest in the subject. That at least twelve per cent. of the deaths from all causes in civilized countries were due to tuberculosis appeared to be conceded on all hands; and when, in connection with this, it was admitted that heredity played a very unimportant part in the propagation of the malady, the very pertinent query as to where and from what sources the disease was acquired by its numerous victims at once suggested itself. The only possible answer was that it spread by contagion, and if, as was admitted, the bacillus was the germ of that contagion, and the bacillus was found in the milk of affected cows and in the lymphatic glands of their bodies, it was difficult to discover the foundation upon which the opponents of the rigid inspection of these articles of diet based their theories. That young children, with their delicate mucous membranes and often imperfect or abused digestion, were the greatest sufferers there was no doubt, but no one was safe from the inroads of this insidious and much-protected malady. The speaker mentioned several instances which had come under his own observation, where there appeared to be no possibility of doubt that the disease had been contracted by children who were consuming milk from affected cows. There was also the often-contested but nevertheless demonstrable fact that in countries where the milk and meat of cows were not articles of food the disease was practically unknown. Many careful experiments had proved beyond a shadow of doubt that the milk and meat of consumptive cows communicated the disease readily to the smaller animals, and if to them, why not to susceptible human subjects? That many men drank milk and ate beef and remained healthy proved nothing. They might be healthy, and they might not. Often the best-looking cow in the herd reacted most violently to the tuberculin test, and the autopsy revealed the presence of the disease. There was no doubt that the same conditions prevailed in regard to the human race, and that many of the healthiest-looking men and women had tubercular lesions localized in their systems, lying in wait for favorable conditions to bring about their development.

That this was really the case was proved by the fact that autopsies conducted on the bodies of suicides and of people who had died from accidents frequently revealed extensive tubercular lesions previously entirely unsuspected. If autopsies were as common in the human subject as they were in cows and oxen, this statement would be even more strongly borne out. The speaker had given the matter a considerable amount of study from the standpoint of legislation, but it was difficult to evolve a satisfactory solution of the question as a whole. Of one thing there could be no doubt, however, viz., that the city of Winnipeg or any other city or town, was perfectly justified in saying to the dairymen, "You shall not give to us or to our children milk from consumptive cows," and to the butchers, "You shall furnish us with meat from healthy animals." He had been criticised somewhat freely by some citizens for the advice which he had given to the Health Committee when asked for an opinion last winter. He had then advised a thorough inspection of all the cows in the dairies, the immediate condemnation and slaughter for rendering, cremation, or burial of those affected with generalized tuberculosis, and the purchase by the city authorities of all which reacted to the tuberculin test. These last, he suggested, should be taken to the country, fattened, and slaughtered under strict veterinary supervision; those in which only trifling and localized lesions were found to be utilized for beef, and those badly affected to be destroyed. Some gentlemen were horrified at the idea of a reputably sensible veterinary surgeon suggesting that the meat of these cows should be utilized, but he had asked them quietly what became of the mortal remains of these animals now; was there some peaceful bovine cemetery on the outskirts of the city where poor, consumptive Crummy was laid to rest, or did they eat her now without the safeguard of the veterinary supervision? No reply to this pointed query was vouchsafed. Dairy cows especially, after having been closely housed and heavily milked, were much more likely to be affected than younger cattle getting more exercise and plenty of fresh air, and he thought that but a small percentage of the ordinary cattle throughout the Province were diseased. They had found in Paris and elsewhere that it was safest to keep the milch-cows in the city dairies for one year only, bringing in fresh and young cows each season, and when one compared the usual ventilation of the average winter cow stable with the amount of fresh air which ought to be supplied, the wisdom of following such a course became apparent. He was a firm believer in the tuberculin test; the number of so-called failures was very small, and there was little doubt that in many of these the characteristic lesions were overlooked. Family cows should all be tested. His own cow was tested, but he observed that the children in his house still had their milk boiled, the result of leaving veterinary periodicals lying round. Mr. McFadyean, he noticed, gave his children boiled milk, and he was a great admirer of Mr. McFadyean. He believed that when circumstances prevented a man going to the front himself, when his capabilities or his opportunities were inadequate for personal scientific investigation, it was a good thing to do as those not in the first flight did in the hunting-field—pick out a good, safe man for a lead, and follow him. Now, in this question of tuberculosis there was no lack of good men to follow. Koch, Nocard, Bang, Crookshank, McCall, McFadyean, and McQueen were all leading the way and sounding the alarm for the members of both branches of the medical profession to fall in and fight the common enemy. The few men who had not as yet seen their way clearly to join in the fray on the right side were likely to find themselves without a leader at all. For his part, he was satisfied with the opinions of such men as those mentioned; they were good enough authorities for him. He trusted that the profession in Manitoba would be a unit on this question, and that when legislation was introduced it would, if in accordance with the undoubted results of scientific and practical investigation, meet with the hearty support of the members of the Association.

